# 1227. Candida Sternal Wound Infections After Cardiac Operations: Uncommon but Deadly

**DOI:** 10.1093/ofid/ofac492.1059

**Published:** 2022-12-15

**Authors:** Jung M Seo, Brian J Louie, Kady Phe, Ravi K Ghanta, Barbara Trautner, Yuriko Fukuta

**Affiliations:** Baylor College of Medicine, Houston, Texas; Baylor College of Medicine, Houston, Texas; Baylor St. Luke's Medical Center, Houston, Texas; Baylor College of Medicine, Houston, Texas; Michael E. DeBakey Veterans Affairs Medical Center / Baylor College of Medicine, Houston, TX; Baylor College of Medicine, Houston, Texas

## Abstract

**Background:**

Sternal wound infections (SWI) are a devastating complication of cardiac surgery. The majority of SWI are bacterial infections; however, *Candida* species are a less common cause and have not been well described. The purposes of this study were (1) to describe clinical characteristics, management and outcomes of *Candida* SWI and (2) to compare the risk factors and outcomes for *Candida* to bacterial SWI.

**Methods:**

Our study reviewed medical records of 41 patients with *Candida* SWI after cardiac surgeries between 2013 - 2020 at our medical center, then compared them to 76 patients with bacterial SWI during the same timeframe via univariate analysis. We defined superficial SWI as positive culture isolates involving the skin or subcutaneous tissues, deep SWI as involving deep soft tissues or bone, and mediastinitis as involving the mediastinum.

**Results:**

Of the 41 *Candida* SWI patients, relevant comorbidities included previous cardiac surgery (46.3%), heart failure (65.9%), and diabetes (58.5%). *Candida* SWI was diagnosed at an average of 123.6 days after cardiac surgery, with the majority being deep SWI (70.7%). *Candida albicans* was most common (70.7%). Bacterial co-infections were found in 53.7% (Table 1). Longer bypass and operative times for the initial cardiac surgery were found to be positively correlated to disease severity. Clinical cure rate after completion of antimicrobial treatment was 100% in superficial SWI, 72.4% in deep SWI and 25.0% in mediastinitis. Overall mortality was 29.3%: 25%, 24.1% and 50.0%, respectively. When compared to bacterial SWI, factors significantly associated with *Candida* SWI included: previous cardiac surgery (46.3% vs. 7.9%, odds ratio (OR): 5.9; 95% confidence interval (CI): 2.2-15.9), heart failure (65.9% vs. 11.8%, OR: 5.6; 95% CI: 2.4-12.9), and >48 hours of postoperative antibiotics (39.0% vs. 3.0%, OR: 9.9; 95% CI: 2.7-35.9). Mortality rates were higher with *Candida* than with bacterial infections (29.3% vs. 1.3%, OR: 22.2, 95% CI 2.8-177.2) (Table 2).

**Figure:**
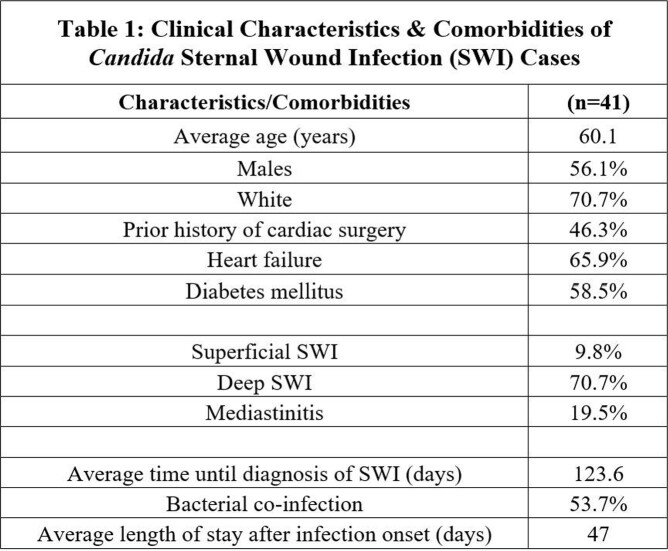

**Figure:**
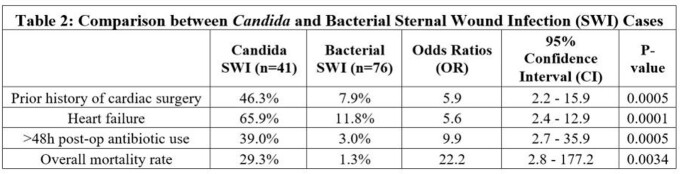

**Conclusion:**

This study showed *Candida* SWI as a serious complication of extensive cardiac surgery with higher mortality rates. Prior history of cardiac surgery and heart failure, prolonged surgeries, and complicated postoperative course were significant risk factors for the development of *Candida* SWI.

**Disclosures:**

**Barbara Trautner, MD, PhD**, Genetech: Advisor/Consultant.

